# Resistance to BTK inhibition by ibrutinib can be overcome by preventing FOXO3a nuclear export and PI3K/AKT activation in B-cell lymphoid malignancies

**DOI:** 10.1038/s41419-019-2158-0

**Published:** 2019-12-04

**Authors:** Isha Kapoor, Yue Li, Arishya Sharma, Huayuan Zhu, Juraj Bodo, Wei Xu, Eric D. Hsi, Brian T. Hill, Alexandru Almasan

**Affiliations:** 1 Department of Cancer Biology, Lerner Research Institute, Cleveland, OH USA; 2 Department of Hematology, the First Affiliated Hospital of Nanjing Medical University, Nanjing, 210029 China; 3 Department of Laboratory Medicine, Institute of Pathology and Laboratory Medicine, Cleveland, OH USA; 4 Department of Hematology and Medical Oncology, Taussig Cancer Institute, Cleveland, OH USA; 5 Department of Radiation Oncology, Taussig Cancer Institute, Cleveland, OH 44195 USA; 6 Case Western Reserve University School of Medicine, Cleveland, OH 44106 USA

**Keywords:** Chronic lymphocytic leukaemia, Chronic lymphocytic leukaemia

## Abstract

Chronic activation of the Bruton’s tyrosine kinase (BTK)-mediated B-cell receptor (BCR) signaling is a hallmark of many B-cell lymphoid malignancies, including chronic lymphocytic leukemia (CLL) and diffuse large B-cell lymphoma (DLBCL). Ibrutinib, an FDA approved, orally administered BTK inhibitor, has demonstrated high response rates, however, complete responses are infrequent and acquired resistance to BTK inhibition can emerge. In this study, we generated ibrutinib-resistant (IB-R) cell lines by chronic exposure of CLL and activated B-cell (ABC)-DLBCL cells to ibrutinib in order to investigate the mechanism of acquired resistance to ibrutinib. IB-R cell lines demonstrated downregulation of FOXO3a and PTEN levels and activation of AKT, with their levels being low in the nuclei of resistant cells in comparison to the sensitive counterparts. Inhibition of PI3K and AKT using idelalisib and MK2206, respectively increased ibrutinib-induced apoptosis in IB-R cells by downregulation of pAKT^473^ and restoring FOXO3a levels, demonstrating the importance of these cell survival factors for ibrutinib-resistance. Notably, the exportin 1 inhibitor, selinexor synergized with ibrutinib in IB-R cells and restored nuclear abundance of FOXO3a and PTEN, suggesting that nuclear accumulation of FOXO3a and PTEN facilitates increase in ibrutinib-induced apoptosis in IB-R cells. These data demonstrate that reactivation of FOXO3a nuclear function enhances the efficacy of ibrutinib and overcomes acquired resistance to ibrutinib. Together, these findings reveal a novel mechanism that confers ibrutinib resistance *via* aberrant nuclear/cytoplasmic subcellular localization of FOXO3a and could be exploited by rational therapeutic combination regimens for effectively treating lymphoid malignancies.

## Introduction

Ibrutinib, an FDA approved, orally administered small-molecule inhibitor that binds covalently to the C481 residue of Bruton’s tyrosine kinase (BTK), has produced remarkable responses in patients with chronic lymphocytic leukemia (CLL) and many subtypes of non-Hodgkin lymphoma (NHL), including mantle cell lymphoma, Waldenstrom macroglobulinemia, and marginal zone lymphoma. The clinical activity of ibrutinib as a single agent in diffuse large B-cell lymphoma (DLBCL) is limited and has preferential benefit to patients with activated B-cell (ABC) subset of DLBCL^1^. However, up to 5% of ibrutinib-treated patients progress with more aggressive ABC-DLBCL, and Richter transformation, a major cause of morbidity and mortality. While curable with R-CHOP treatment in the majority of DLBCL patients, up-to one-third of those patients develop relapsed/refractory disease^2–4^. BTK is a critical kinase in the B-cell receptor (BCR) pathway that drives BCR signaling cascade leading to activation of downstream NF-κB and phosphatidylinositol-3-kinase (PI3K) pro-survival pathways in CLL, indolent forms of NHL, and ABC-DLBCL^5^. Despite encouraging results, responses to ibrutinib are variable/partial, often leading to drug resistance and aggressive relapse in the clinic, often from acquisition of mutations in BTKC481S or phospholipase C gamma 2 (PLCG2)^6,7^.

Recent studies showed constitutive activation of the PI3K/AKT pathway in 25–52% of DLBCL patients. The non-redundant role downstream of PI3K, particularly of the PI3Kδ isoform has been implicated as a central mechanism for relaying cell survival, adhesion, and proliferative signals^8,9^. Additional studies have correlated overexpression of phosphorylated Akt (pAKT) with significantly poorer progression-free survival in ~1/4th of DLBCL patients^10^. Loss of PTEN (phosphatase and tensin homolog), a major negative regulator of the PI3K/AKT signaling is significantly associated with advanced disease, chemotherapy resistance, and poor survival in DLBCL patients with DLBCL with AKT hyperactivation^11–13^.

Forkhead box O (FOXO) transcription factors are tumor suppressors that serve as critical regulators of cellular proliferation, differentiation, cell-cycle arrest, and apoptosis^14^. FOXO3a is a *bona fide* tumor suppressor in lymphoid peripheral tissues and its inactivation is essential for proliferation of immune cells, as shown in B- and T-lymphocytes^15^. AKT acts as an important upstream regulator of FOXO3a, directly phosphorylating FOXO3a, leading to its sequestration in the cytoplasm and consequently its degradation. Thus, less FOXO3a protein accumulates in the nuclei to drive transcriptional activation of target genes involved in apoptosis, including *pten* and *bim*^16^. Phosphorylated, cytoplasmic FOXO3a is associated with malignant transformation and poor prognosis in patient-derived mantle cell lymphoma^15^. Reactivation of FOXO3a function with a nuclear export inhibitor had a profound effect on cell viability, consistent with its nuclear accumulation^15^. Therefore, exploration of the FOXO3a/PTEN/AKT signaling and its involvement in apoptosis in ibrutinib-resistant (IB-R) B-cell malignancies is of significant clinical relevance.

It has been shown that CLL and DLBCL patients acquire resistance to ibrutinib through mutations in BTK and its substrate phospholipase C gamma 2 (PLCG2)^6,17^, MYD88^18^, and CARD11^17^ following prolonged treatment. In addition to acquisition of these mutations, other mechanisms can confer treatment resistance to ibrutinib, such as upregulation of druggable survival pathways^8^ or clonal evolution due to other genetic alterations^19,20^. Such mechanisms may be overcome by rational therapeutic combinations of targeted agents that block adaptive pathways promoting drug resistance. Therefore, we investigated the underlying molecular signatures of IB-R in sensitive *vs* acquired IB-R cells following chronic exposure to ibrutinib. By comparing sensitive vs acquired IB-R cells, we have defined IB-R as FOXO3a/PTEN/AKT-dependent in CLL and DLBCL in the absence of BTK or PLCG2 mutations. Our data reveal novel mechanistic insights into the role of FOXO3a subcellular localization in IB-R cells and provide a rationale for combination strategies to overcome it in lymphoid malignancies by restoring nuclear accumulation of FOXO3a.

## Results

### Acquired ibrutinib resistance following chronic exposure to ibrutinib leads to deregulation of the FOXO3a/PTEN/AKT axis

**Fig. 1:**
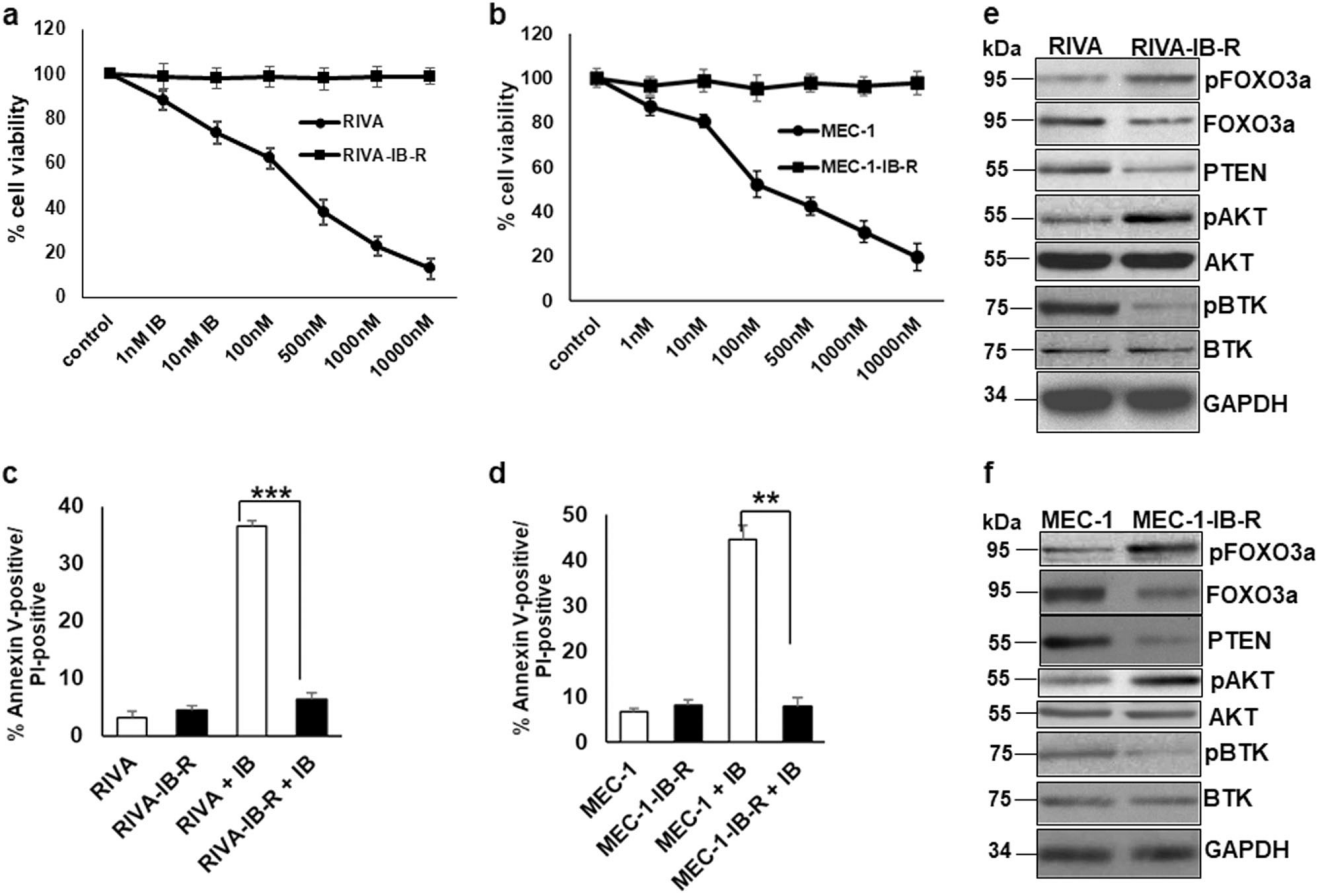
Acquired resistance to ibrutinib leads to decreased FOXO3a and PTEN levels and activation of AKT. **a**, **b** RIVA and MEC-1 cells were treated with the indicated concentrations of ibrutinib for 72 h and cell viability was determined by the MTS assay. Control cells were treated with DMSO. **c**, **d** Cell death analysis in parental (RIVA, MEC-1) and ibrutinib-resistant derivatives (RIVA-IB-R, MEC-1IB-R) in response to 24 h ibrutinib treatment determined by Annexin-V/PI staining. All data are expressed as mean ± S.D. of percentage of cell death. Standard deviation (SD) is indicated as error bars (*N* *=* 3). (*p *<* 0.05, **p *<* 0.01, ***p *<* 0.001). **e**, **f** Expression levels of pFOXO3a^Ser253^, FOXO3a, pAKT^Ser473^, AKT and PTEN in whole cell extracts of untreated parental and IB-R RIVA and MEC-1 cell lines. GAPDH was used as a loading control.

Ibrutinib-resistant (IB-R) ABC-DLBCL (RIVA, TMD8) and CLL (MEC-1) cell lines were generated by culturing the parental cell line in vitro with progressively increasing concentrations of ibrutinib. Cell viability analysis by MTS assay demonstrated a high sensitivity to increasing concentrations of ibrutinib administered for 72 h in the parental cell lines, with an IC_50_ of 85 nM for RIVA, 23 nM for TMD8, and 109 nM for MEC-1 cells. These IB-R-derivative cells were resistant to ibrutinib at concentrations 5-fold higher than the IC_50_ of the parental cells (Fig. 1a, b and Supplementary Fig. S1a). Similarly, Annexin-V/PI staining showed ~35% increase in cell death in RIVA and TMD8 and ~45% in MEC-1 cells (Fig. 1c, d and Supplementary Fig. S1b), but not in IB-R variants after 24 h ibrutinib treatment.

To investigate the mechanism of IB-R, we examined the expression patterns of tumor suppressor proteins FOXO3a and PTEN in parental and resistant cells. Immunoblot analyses show that the levels of FOXO3a and PTEN were low in resistant (RIVA-IB-R, TMD8-IB-R, and MEC-1-IB-R) as compared to parental cells while the levels of pFOXO3a^Ser253^ phosphorylation were conspicuously higher in resistant cells (Fig. 1e, f and Supplementary Fig. S1c). Additionally, levels of pAKT (AKT^Ser473^) were upregulated in RIVA-IB-R and MEC-1-IB-R cells in comparison to parental cells. Importantly, pBTK (BTK^Tyr223^) levels were diminished as an indication that ibrutinib can still block BTK activation in IB-R cells. Targeted sequencing analysis performed for the C481S mutation in BTK and R665W, S707Y and L845F mutations in PLCG2 in these two pairs of cell lines confirmed absence of acquisition of hotspot mutations in IB-R cells. Taken together, these findings indicate the importance of FOXO3a/PTEN/AKT axis in mediating IB-R.

### Ibrutinib inhibits AKT and activates FOXO3a and PTEN in CLL and ABC-DLBCL

**Fig. 2:**
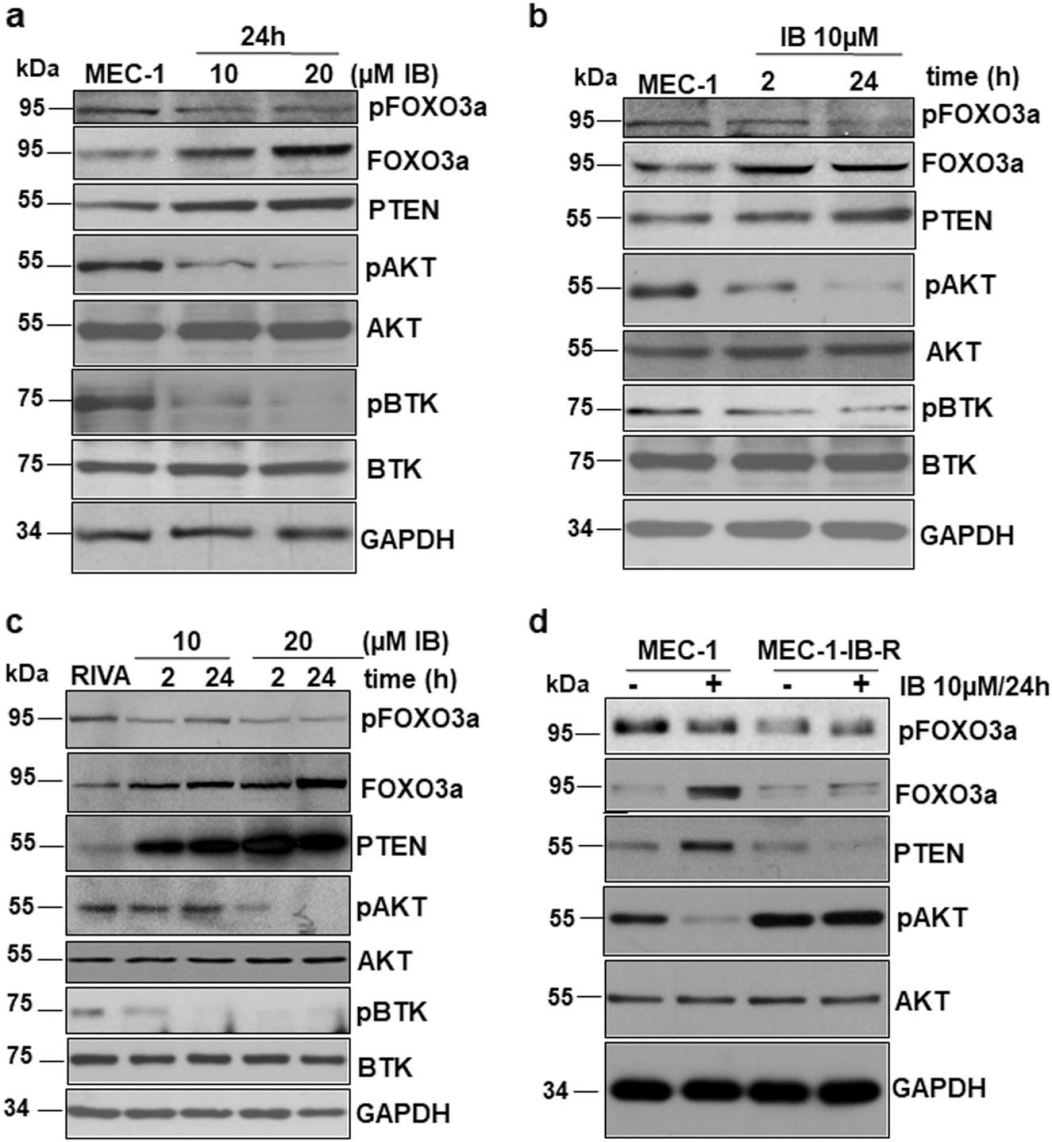
Ibrutinib treatment upregulates FOXO3a/PTEN signaling and inhibits AKT. **a**–**c** MEC-1 and RIVA cells were treated with ibrutinib in a dose-and time-dependent manner as indicated and immunoblotted for PTEN, pAKT^Ser473,^ pFOXO3a^Ser253^, AKT, and FOXO3a. GAPDH was used as a loading control. **d** MEC-1 parental and IB-R cells were treated with or without ibrutinib (10 µM) for the indicated time. Expression levels of pAKT^Ser473^, FOXO3a and PTEN were determined by immunoblotting. GAPDH was used as a loading control.

Since IB-R following chronic exposure to ibrutinib resulted in low FOXO3a and PTEN levels and activation of AKT, we examined the effect of acute ibrutinib treatment on this pathway in ibrutinib sensitive CLL and ABC-DLBCL cell lines. Parental MEC-1 cells demonstrated upregulation of FOXO3a and PTEN levels with ibrutinib treatment in a dose- and time-dependent manner (Fig. 2a, b). pAKT^Ser473^ levels were concomitantly reduced together with pFOXO3a^Ser253^ unlike IB-R cells. Similar results were observed in RIVA parental cells (Fig. 2c).

Next, the effect of ibrutinib treatment on the expression of FOXO3a/PTEN/AKT signaling axis was examined in parental *vs* resistant cells. Immunoblot analyses indicated downregulation of pAKT in MEC-1 parental compared to resistant cells (Fig. 2d). Notably, the levels of FOXO3a and PTEN could not be rescued in MEC-1-IB-R cells even after ibrutinib treatment to comparable levels in parental cells (Fig. 2d), indicating the plausible role of FOXO3a/PTEN/AKT signaling axis in mediating IB-R.

### Ibrutinib treatment regulates FOXO3a phosphorylation, nuclear translocation, and transcriptional activation of *pten* and *bim*

**Fig. 3:**
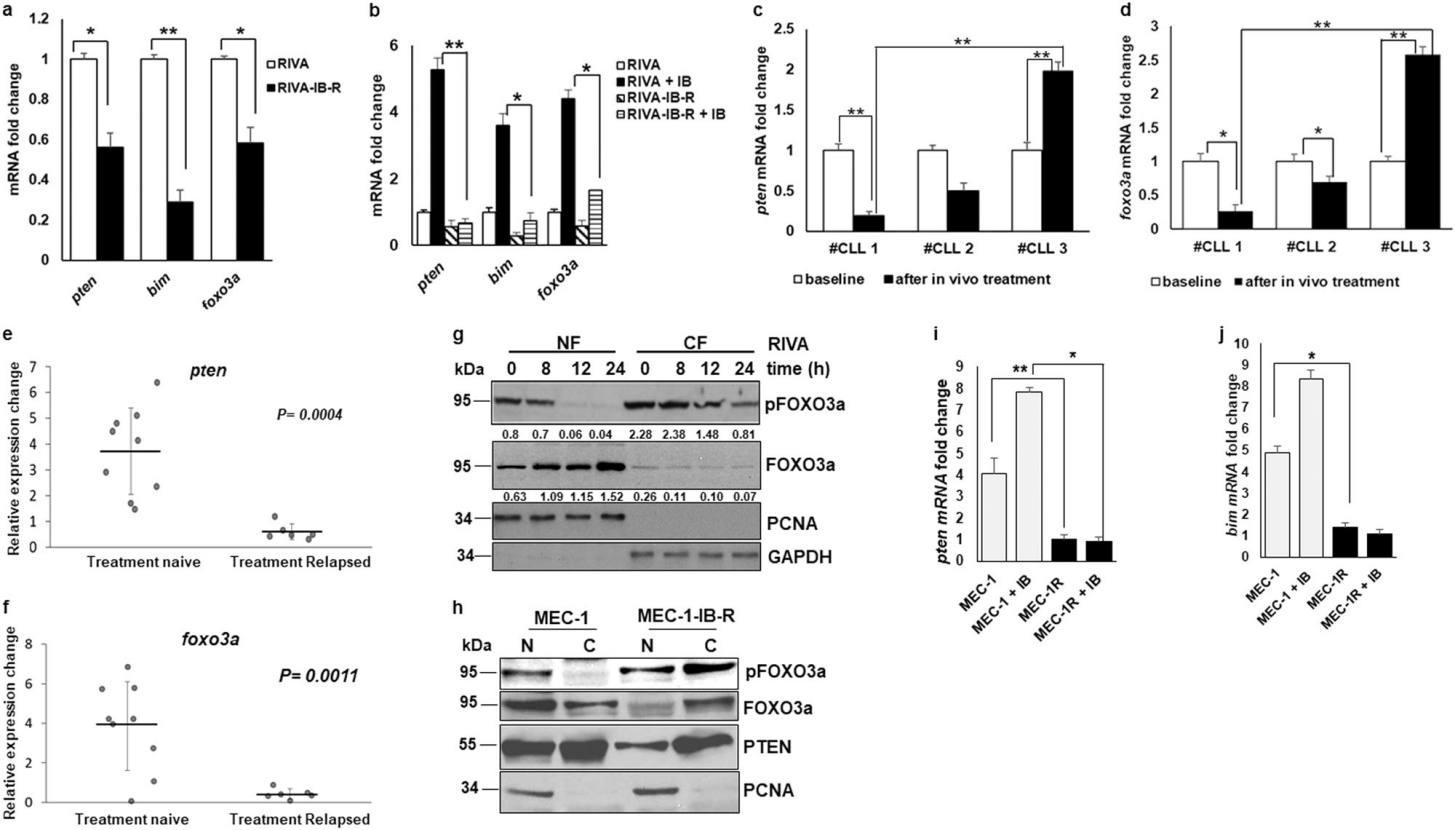
Ibrutinib treatment regulates FOXO3a phosphorylation, nuclear translocation, and transcriptional activation of *pten* and *bim*. **a** mRNA fold change of *pten*, *bim*, and *foxo3a* in parental *vs* IB-R RIVA cells after culture in the absence of ibrutinib for 72 h (*p *<* 0.05, **p *<* 0.01). SD is indicated as error bars (*N* *=* 3). **b** mRNA fold change of *pten*, *bim* and *foxo3a* in parental *vs* IB-R RIVA cells with or without ibrutinib (10 µM). **c** *pten* mRNA fold change was analyzed in primary cells obtained from 3 paired CLL patients’ samples pre- and post-ibrutinib treated in the clinic. SD is indicated as error bars (*N* *=* *1; triplicates of 1 experiment*). **d** *foxo3a* mRNA fold change was analyzed in primary cells obtained from 3 paired CLL patients’ samples pre- and post-ibrutinib treated in the clinic. SD is indicated as error bars (*N* *=* *1, as in c*). **e**, **f** relative expression changes in *pten* and *foxo3a* mRNA was analyzed in primary cells obtained from 9 treatment naive *vs* 5 treatment relapsed CLL patients’ samples after in vitro treatment with ibrutinib. Man–Whitney nonparametric analysis was performed to compare them. Two-sided *p* value for *pten* is 0.0004 and *foxo3a* is 0.0011. **g** Expression levels of pFOXO3a ^Ser253^, FOXO3a, and PTEN in nuclear/ cytoplasmic fractions of parental RIVA cells after treatment with ibrutinib (10 µM) for the indicated time points. PCNA and GAPDH were used as a nuclear fraction and cytoplasm loading controls, respectively. **h** Expression levels of pFOXO3a^Ser253^, FOXO3a, and PTEN in nuclear/cytoplasmic fractions of untreated parental and IB-R MEC-1 cells after culture in the absence of ibrutinib for 48 h. PCNA was used as a nuclear fraction loading control. MEC-1 parental and IB-R cells were treated with or without ibrutinib (10 µM) for 24 h followed by the ChIP assay. **i**, **j** qRT-PCR was performed for the *pten* and *bim* promoters. (*p *<* 0.05, **p *<* 0.01). SD is indicated as error bars (*N* *=* 2).

Since IB-resistance resulted in downregulation of FOXO3a and PTEN levels following chronic exposure to ibrutinib, we investigated the underlying cause of the decrease in the levels of these proteins in IB-R cells. qRT-PCR analyses indicated that both *foxo3a and pten* mRNA levels were decreased by 2-fold and *bim* mRNA levels were decreased by 3.5-fold in RIVA-IB-R cells (Fig. 3a). Similar results were obtained in MEC-1-IB-R cells (Supplementary Fig. S2a), indicating that reduced FOXO3a and PTEN levels in resistant cells could be attributed to the decreased mRNA levels. Similar results were obtained in RIVA-IB-R and TMD8-IB-R cells after acute treatment with ibrutinib (Fig. 3b and Supplementary Fig. S1d and e). In contrast, qRT-PCR analyses in MEC-1 and RIVA cells revealed that *pten* and *foxo3a* mRNA levels were increased both in a time-and dose-dependent manner (Supplementary Fig. S2b, c).

Next, qRT-PCR analyses in 3 paired CLL patient samples (ibrutinib resistant #CLL 1, partial remission #CLL 2, and ibrutinib sensitive #CLL 3) pre- and post-ibrutinib treatment in the clinic revealed an increase in *pten* and *foxo3a* in #CLL 3 sample after ibrutinib treatment in contrast to #CLL 1 (Fig. 3c and d). Additionally, in vitro ibrutinib treatment of naive vs treatment-relapsed CLL patient samples revealed an increase in both *pten* and *foxo3a* mRNA levels in treatment naive in contrast to relapsed patients (Fig. 3e and f).

Accumulating evidence indicates that the tumor suppressor functions of FOXO3a are mediated by its nuclear localization, which, in turn, is regulated by pSer253 phosphorylation^21^. Accumulated FOXO3a in the nucleus binds to promoters containing a consensus sequence to enhance the transcription of various effectors in apoptosis, such as *bim*, *pten*, and *puma*^22^. Therefore, since ibrutinib treatment in sensitive cells resulted in upregulation of FOXO3a levels in a time- and dose-dependent manner, we hypothesized that FOXO3a nuclear localization may be regulated by ibrutinib leading to transcriptional activation of *pten* and *bim* to induce apoptosis. Immunoblotting of FOXO3a in nuclear/cytoplasmic fractions of ibrutinib-treated RIVA cells revealed that levels of FOXO3a increased in the nucleus while those of its phosphorylated form decreased in the nuclear fraction (Fig. 3g).

Next, we postulated that reduced levels of nuclear FOXO3a might play a critical role in mediating IB-R. We examined the levels of FOXO3a in MEC-1 parental and IB-R cells in the cytoplasmic and nuclear compartments. Interestingly, immunoblot analyses indicated low levels of FOXO3a in the nuclear fraction of resistant in comparison to parental cells (Fig. 3h). PTEN is known to be expressed in both the cytoplasm and nucleus and nuclear localized PTEN has tumor-suppressive functions^23^. Consistently, we found reduced levels of PTEN in the nuclei of resistant in comparison to parental cells (Fig. 3h).

Next, to examine whether ibrutinib-induced FOXO3a nuclear accumulation could influence transcriptional activation of *pten* and *bim* in sensitive *vs* MEC-1-IB-R cells, we performed chromatin immunoprecipitation (ChIP). Indeed, ibrutinib treatment in MEC-1 cells enhanced FOXO3a binding to the *pten* promoter (Fig. 3i), thus enhancing its transcription. In contrast, in MEC-1-IB-R cells FOXO3a binding to the *pten* promoter was abrogated (Fig. 3i). Similar results were obtained with the *bim* promoter (Fig. 3j). Taken together, these results indicate that ibrutinib induces FOXO3a activation and nuclear translocation which, in turn, led to its binding to the *pten* and *bim* promoters to activate their transcription and induce apoptosis.

### FOXO3a-upregulated PTEN expression antagonizes AKT to induce apoptosis

**Fig. 4:**
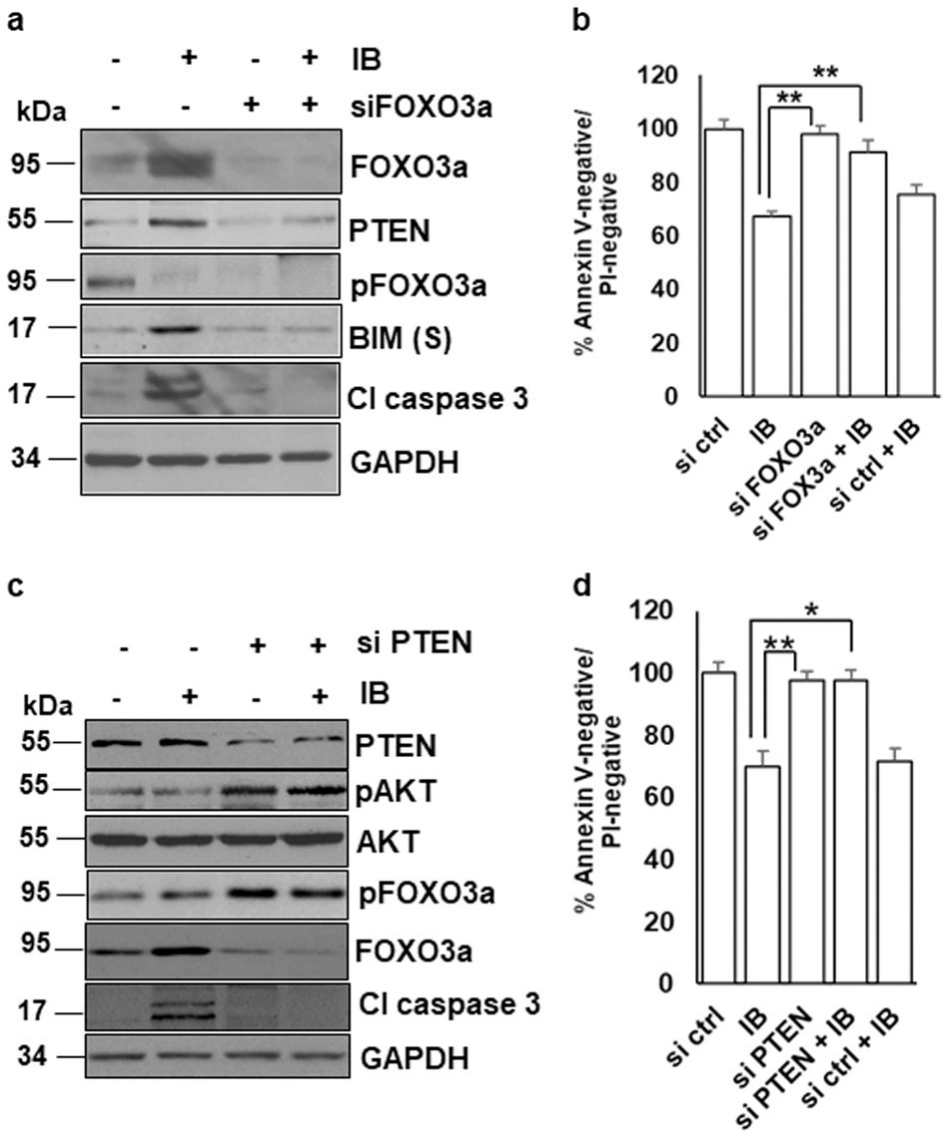
Ibrutinib-induced FOXO3a upregulates PTEN that antagonizes AKT to induce apoptosis. **a** RIVA cells were transfected with siFOXO3a and siControl and treated with ibrutinib (10 µM) for 24 h. Expression levels of FOXO3a, PTEN, pFOXO3a^Ser253^, BIM, and cleaved Caspase 3 were determined by immunoblotting with the indicated antibodies. GAPDH was used as a loading control. **b** Cell viability was determined by Annexin V-PI staining. Control cells were treated with DMSO. (*p *<* 0.05*,* **p *<* 0.01). SD is indicated as error bars (*N* *=* 3). **c** MEC-1 cells were transfected with siPTEN and siControl and treated with ibrutinib (10 µM) for 24 h. Expression levels of PTEN, pAKT, AKT, pFOXO3a^Ser253^, FOXO3a, and cleaved Caspase 3 were determined by immunoblotting with the indicated antibodies. GAPDH was used as a loading control. **d** Cell viability was determined by Annexin V-PI staining. Control cells were treated with DMSO. (*p *<* 0.05). SD is indicated as error bars (*N* *=* 3).

To confirm the role of FOXO3a-dependent transcriptional activation of *pten* and *bim* in ibrutinib-induced apoptosis, expression of FOXO3a was silenced with siRNA in RIVA cells treated with ibrutinib. FOXO3a silencing markedly reduced the expression of PTEN, caspase-3 cleavage, and the short isoform of pro-apoptotic BIM(S) protein levels (Fig. 4a). Cell viability analysis by Annexin V-PI staining in these cells also confirmed inhibition of ibrutinib-induced apoptosis with FOXO3a silencing (Fig. 4b). To clarify whether FOXO3a-dependent downregulation of BIM could regulate ibrutinib-induced apoptosis, expression of BIM was silenced with siRNA in RIVA cells treated with ibrutinib leading to markedly decreased apoptosis (Supplementary Fig. S3).

To clarify whether upregulation of PTEN could indeed modulate AKT by antagonizing AKT *via* feedback inhibition and consequently apoptosis, PTEN expression was silenced in MEC-1 cells with siRNA. PTEN knock-down failed to antagonize AKT, as demonstrated by increased AKT phosphorylation leading to inhibition of ibrutinib-induced apoptosis as revealed by reduced caspase-3 cleavage and Annexin V-PI staining (Fig. 4c, d). From these results, we concluded that ibrutinib-induced activation of FOXO3a and apoptosis were critically regulated by PTEN and BIM.

### PI3K/AKT inhibition upregulates FOXO3a level and sensitizes IB-R cells

**Fig. 5:**
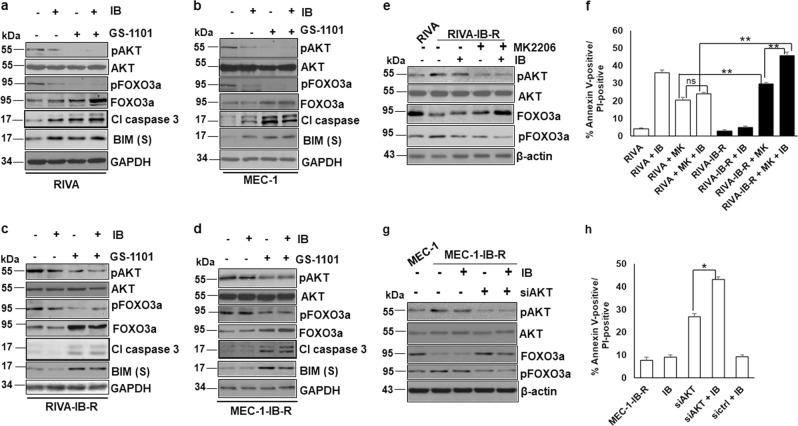
PI3K/AKT inhibition upregulates FOXO3a levels and sensitizes IB-R cells. **a**, **b** RIVA and MEC-1 cells were treated with ibrutinib (10 µM) and GS-1101 (5 µM) alone or in combination for 24 h. Expression levels of pAKT, AKT, pFOXO3a^Ser253^, FOXO3a, cleaved Caspase 3 and BIM were determined by immunoblotting. GAPDH was used as a loading control. **c**, **d** RIVA-IB-R and MEC-1-IB-R cells were treated with ibrutinib (10 µM) and GS-1101 alone or in combination for 24 h. Expression levels of pAKT, AKT, pFOXO3a^Ser253^, FOXO3a and cleaved Caspase 3 were determined by immunoblotting. GAPDH was used as a loading control. **e** RIVA-IB-R cells were treated with ibrutinib (10 µM) and AKTi (MK2206, MK) (5 µM for 48 h) alone or in combination. Expression levels of pAKT, AKT, pFOXO3a^Ser253^, and FOXO3a were determined by immunoblotting. β-actin was used as a loading control. First lane indicates the baseline expression of proteins in parental RIVA cells. **f** RIVA and RIVA-IB-R cells were treated with MK2206 (5 µM) and ibrutinib (10 µM) either alone or in combination for 24 h. Cell viability was determined by Annexin V-PI staining. Control cells were treated with DMSO. (*p *<* 0.05). SD is indicated as error bars (*N* *=* 3). **g** MEC-1-IB-R were transfected with siAKT and treated with ibrutinib (10 µM) for 24 h. Expression levels of pAKT^473^, AKT, pFOXO3a^Ser253^ and FOXO3a were determined by immunoblotting. β-actin was used as a loading control. First lane indicates the baseline expression of proteins in MEC-1 cells. **h** MEC-1-IB-R cells were transfected with siAKT or siControl and treated with ibrutinib (10 µM) for 24 h. Cell viability was determined by Annexin V-PI staining. Control cells were treated with DMSO. (*p < 0.05). SD is indicated as error bars (*N* *=* 3).

Given the dependency of IB-R cells on the FOXO3a/PTEN/AKT signaling, we investigated whether these cells could be sensitized to ibrutinib by targeting the PI3K/AKT pathway. RIVA and MEC-1 cells and their resistant derivatives were treated with the PI3Kδ and AKT inhibitors, GS-1101 and MK2206, respectively, in combination with ibrutinib for 24 h. Immunoblot analyses indicated downregulation of pAKT together with pFOXO3a and concomitant upregulation of FOXO3a levels in both RIVA and MEC-1 (Fig. 5a, b). Notably, pro-apoptotic BIM levels were upregulated together with proteolytic cleavage of caspase 3, indicating induction of apoptosis (Fig. 5a, b). Similar results were observed in RIVA-IB-R and MEC-1-IB-R cells following the GS-1101 + ibrutinib combination (Fig. 5c, d).

Next, AKT was inhibited either pharmacologically by MK2206 or genetically by siRNA-mediated knock-down together with ibrutinib treatment. AKT inhibition in RIVA-IB-R (Fig. 5e) led to downregulation of AKT phosphorylation. FOXO3a phosphorylation was also reduced with simultaneous upregulation of FOXO3a. Moreover, cell viability analysis by Annexin-V/PI staining in these cells also confirmed increase in ibrutinib-induced apoptosis with AKT inhibition in RIVA-IB-R cells (Fig. 5f). Similar results were also obtained with AKT inhibition in MEC-1-IB-R and TMD8-IB-R cells (Supplementary Figs. S4a and S1f). Notably, siRNA-mediated knockdown of AKT in MEC-1-IB-R (Fig. 5g, h) and RIVA-IB-R cells also resulted in increase in ibrutinib-induced apoptosis with AKT inhibition (Supplementary Fig. S4b). Additionally, siRNA-mediated knockdown of AKT in MEC-1 and RIVA cells treated with ibrutinib indicated no significant dependence of parental cells on AKT for cell survival (Supplementary Fig. S4c and d). Together, these results indicate the dependency of IB-R cells on FOXO3a/PTEN/AKT signaling pathway and that inhibition of AKT phosphorylation/activation increases ibrutinib-induced apoptosis in IB-R cells *via* restoring FOXO3a function.

### Selinexor synergizes with ibrutinib and restores FOXO3a nuclear accumulation in IB-R cells

**Fig. 6:**
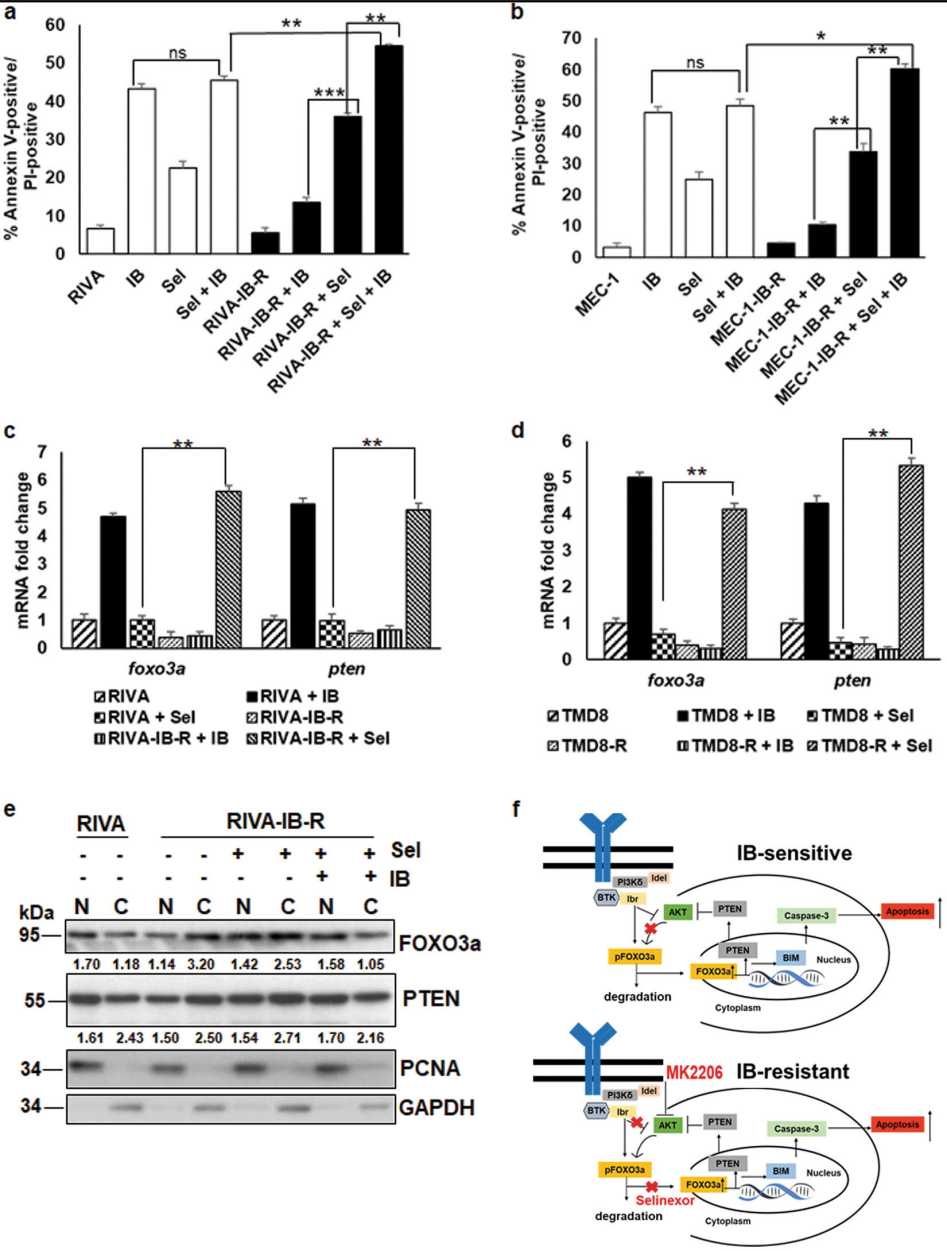
Selinexor synergizes with ibrutinib by restoring FOXO3a nuclear accumulation in IB-R cells. **a**, **b** RIVA/ RIVA-IB-R and MEC-1/ MEC-1-IB-R cells were treated with ibrutinib and selinexor either alone or in combination for 24 h. Cell viability was determined by Annexin V-PI staining. Control cells were treated with DMSO. (*p *<* 0.05*,* **p *<* 0.01, ***p *<* 0.001). SD is indicated as error bars (*N* *=* 3). **c**, **d** mRNA fold change of *foxo3a and pten* in parental *vs* IB-R RIVA and TMD8 cells treated with either ibrutinib or selinexor (0.5 µM). **e** RIVA-IB-R cells were treated with selinexor (500 nM) and ibrutinib (1 µM) alone or in combination. Expression levels of FOXO3a and PTEN were examined in nuclear and cytoplasmic fractions. PCNA and GAPDH were used as loading controls for the nuclear and cytoplasmic fractions, respectively. First two lanes indicate baseline expression of proteins in RIVA cells. Model of acquired IB-R mechanism. **f** Reduced FOXO3a phosphorylation (FOXO3a^Ser253^) followed by its nuclear accumulation after ibrutinib treatment increases transcriptional activation of PTEN and BIM, leading to apoptosis and AKT inactivation in IB-sensitive cells (upper panel). In IB-R cells, pFOXO3a^Ser253^ is upregulated resulting into its reduced nuclear accumulation and transcriptional inhibition of PTEN and BIM. Selinexor overcomes IB-R by restoring nuclear accumulation of FOXO3a leading to transcriptional activation of PTEN and BIM thus, leading to apoptosis. Idelalisib and MK2206 overcome IB-R by exploiting IB-R cells’ dependency on PI3K/AKT pro-survival signaling (lower panel).

Having discovered that IB-R leads to reduced FOXO3a levels in the nucleus, while ibrutinib treatment in sensitive, parental cells resulted in its nuclear accumulation, we hypothesized that restoration of FOXO3a in the nuclei of IB-R cells by a nuclear export inhibitor could overcome FOXO3a/PTEN-dependent resistance. Additionally, accumulating studies also suggest that deregulation of the nucleo-cytoplasmic shuttling of tumor suppressor proteins, including FOXO3a have a direct role in promoting aberrant cell survival, tumor progression, and drug resistance in various hematological malignancies^24^. Selinexor (KPT-330) is a selective nuclear export inhibitor of exportin-1 (CRM1/XPO1) in various hematological malignancies, including CLL. Selinexor targets XPO1 and restores the subcellular localization of its various dysregulated tumor suppressor cargos, including FOXO3a^25^. Therefore, we investigated whether treatment with selinexor alone or in combination with ibrutinib could rescue nuclear levels of FOXO3a/PTEN and sensitize IB-R cells. Thus, RIVA and IB-R-derivate cells were treated with selinexor alone or in combination with ibrutinib for 72 h. Cell viability analysis by Annexin-V/PI staining in these cells confirmed that combination of selinexor and ibrutinib increases ibrutinib-induced apoptosis in RIVA-IB-R cells (Fig. 6a). Similar results were also obtained with MEC-1 parental and IB-R-derivative cells treated with either selinexor alone or in combination with ibrutinib for 72 h (Fig. 6b). Additionally, cell viability analysis by MTS assay revealed that RIVA cells display antagonism (CI > 1) at lower concentrations of selinexor, while RIVA-IB-R cells exhibit synergy (CI < 1) at majority of drug concentrations tested (Supplementary Fig. S5a, b). Similar results were observed in MEC-1 *vs* MEC-1-IB-R cells (Supplementary Fig. S5c, d). Taken together, our results indicate that although these two IB-R cells displayed different degrees of response to selinexor as a single agents, synergy was observed for both leading to greater sensitivity to sel + IB combination in comparison to parental cells.

Further, qRT-PCR analysis revealed increase in both *foxo3a* (~5-fold) and *pten* (~4-fold) mRNA levels in RIVA-IB-R cells treated with selinexor (Fig. 6c). Similar results were also obtained in TMD8-IB-R cells treated with selinexor (Fig. 6d). Finally, we analyzed the subcellular localization of FOXO3a and PTEN in isolated nuclear-cytoplasmic fractions of RIVA-IB-R cells treated with either selinexor or ibrutinib, alone or in combination for 24 h. The abundance of FOXO3a (~1.4-fold) and PTEN (~1.1-fold) increased in the nuclear compartment upon treatment with both the selinexor alone and in combination with ibrutinib in RIVA-IB-R cells (Fig. 6e), indicating that accumulation of FOXO3a and PTEN in the nucleus facilitates sensitization of IB-R cells.

## Discussion

AKT hyperactivation has been identified as an important drug resistance mechanism in many tumors, including B-cell malignancies^10,26,27^. Our study uniquely characterized the development of acquired IB-R by demonstrating that FOXO3a is a critical target of inactivation. FOXO3a is a *bona fide* tumor suppressor in B-cell malignancies and regulates diverse cellular activities, including cell-cycle arrest and apoptosis by transcriptional activation of target genes, such as *bim*, *puma*, *p27*, and *pten*^14^. The present study provides evidence that ibrutinib-induced FOXO3a promotes apoptosis in a PTEN- (Fig. 4c and d) and BIM-dependent manner (Supplementary Fig. S3a and b). Importantly, aberrant nuclear/ cytoplasmic translocation of FOXO3a in IB-R cells leads to transcriptional inhibition of *pten* and *bim*. We found that ibrutinib-induced FOXO3a nuclear accumulation and activation triggers apoptosis by transcriptional activation of *pten* and *bim,* *via* their increased promoter binding by FOXO3a (Fig. 3i, j), consistent with investigations demonstrating that constitutively expressed nuclear forms of FOXO3a activate cell death by transactivation of pro-apoptotic BH3-only proteins^21^. In addition, silencing of FOXO3a greatly reduced ibrutinib-induced apoptosis mediated by downregulation of PTEN and BIM. Importantly, we show that silencing of PTEN abrogated the effects of ibrutinib, as demonstrated by AKT activation. Together, our findings demonstrate that ibrutinib-induced FOXO3a nuclear accumulation and activation leading to PTEN-dependent inhibition of AKT is a determinant of ibrutinib-induced apoptosis. Therefore, restoring expression of FOXO3a and its nuclear reactivation represents an attractive therapeutic strategy to overcome IB-R.

Several studies have demonstrated expression of phosphorylated and cytoplasmic FOXO3a, both being linked to FOXO3a inactivation and poor prognosis in several cancers^26,28–30^, including mantle cell lymphoma^15^. Post-translational modifications of FOXO3a, including phosphorylation by AKT leads to FOXO3a sequestration in the cytoplasm and its inactivation^21^. Consistently, our data revealed for the first-time increased expression of phosphorylated and cytoplasmic FOXO3a in IB-R cells. Additionally, unlike IB-R cells, ibrutinib treatment in sensitive cells resulted in decreased phosphorylation of FOXO3a, which coincided with the depletion of the cytoplasmic FOXO3a pool and its accumulation in the nucleus. Our data demonstrating sustained high AKT phosphorylation in IB-R cells suggests amplification of alternative signaling pathways as adaptive mechanism *via* compensatory activation of pro-survival PI3K/AKT signaling. This is in line with the complex nature of the resistance mechanism, as previously reported in IB-R mantle cell lymphoma^27^, venetoclax (ABT-199)-resistant^8^, or fludarabine-resistant^31^ B-cell lymphoid malignancies, where inhibition of key determinants represented a powerful approach to overcome drug resistance and induce apoptosis. Consistently, AKT activation was, indeed, elevated in our IB-R cells and FOXO3a/ PTEN expression was significantly downregulated, thus making IB-R more sensitive to AKT inhibition and induction of apoptosis.

A central role of PI3K signaling in the development of IB-R in a patient-derived xenograft model has been proposed recently while a possible mechanism is not fully elucidated^32^ and targeting upstream regulators of AKT holds a therapeutic potential. Loss of PTEN is a well-defined mechanism of upregulation of PI3K/AKT; as loss of PTEN does not offer a practical therapeutic option, we did not pursue this avenue^33^. Several PI3K inhibitors are being tested in clinical trials, including pan-PI3K inhibitors that demonstrate great efficacy but also produce adverse toxicity^34^. As such, selective PI3K isoform specific inhibitors are of interest^35,36^. Pro-survival signals are specifically mediated by the delta isoform (PI3Kδ) of PI3K that is mainly expressed on B cells. The PI3Kδ inhibitor idelalisib (GS-1101) was approved by the FDA for treating relapsed CLL, small lymphocytic lymphoma (SLL) and follicular lymphoma (FL)^37,38^. Interestingly, our results showed that PI3Kδ inhibition by idelalisib sensitized RIVA-IB-R cells to apoptosis, suggesting that idelalisib is a well-tolerated salvage drug for Richter transformation. Consistently, our preclinical observations are in line with the clinical efficacy of idelalisib reported in IB-R patients^39^, thus indicating that a combination of idelalisib with ibrutinib is a promising clinically amenable and effective alternative. Additionally, ongoing clinical trials of ibrutinib with improved PI3K inhibitors suggest that a dual BCR pathway blockade is tolerable and efficacious in relapsed or refractory CLL and mantle cell lymphoma^40^.

Our data demonstrated that aberrant nucleo-cytoplasmic shuttling of FOXO3a is a major target of inactivation in IB-R cells. Thus, using a nuclear export inhibitor, selinexor together with ibrutinib resulted in synergistic induction of apoptosis suggesting its therapeutic potential to prevent expansion of IB-R subclones. The ability of selinexor as a single agent to induce apoptosis in IB-R cells closely correlates with the nuclear FOXO3a expression levels, suggesting that reactivation of FOXO3a function enhances the efficacy of ibrutinib to overcome resistance. Consistently, previous studies have shown that selinexor exhibits pro-apoptotic activity against CLL cells *via* inhibition of nuclear export of tumor suppressor proteins^41^ and in vitro efficacy in IB-R CLL^25^, thus supporting our preclinical findings. Additionally, our results suggest the ability of selinexor as a single agent to block adaptive signaling responses in resistant subclones to overcome drug resistance in a manner independent of BTK kinase activity. Indeed, a small molecule inhibitor of the transcriptional repressor BCL-6 has been shown to induce cell death in DLBCL^42^, reinforcing the fact that transcription factors, including FOXO3a can be therapeutically targeted, and represent a potential approach to elicit responses in acquired IB-R.

In summary, our findings provide novel molecular insights into IB-R mechanisms and support a link between aberrant nucleo-cytoplasmic shuttling of FOXO3a in IB-R cells and the ability of selinexor to promote FOXO3a nuclear translocation and reactivation to overcome drug resistance and induce apoptosis (Fig. 6f). Importantly, an idelalisib ibrutinib combination leading to induction of apoptosis exploits the resistant cell’s dependency on FOXO3a/PTEN axis by downregulating AKT and restoring FOXO3a levels (Fig. 6f). Thus, the FOXO3a/PTEN/AKT-axis emerges as a critical determinant of acquired IB-R in CLL and DLBCL. Reduced nuclear FOXO3a downregulates PTEN and BIM and promotes pro-survival AKT activation in IB-R cells. We propose a combination of selinexor with ibrutinib to restore nuclear accumulation of FOXO3a, PI3K/AKT inhibitors, and ibrutinib as rational combination strategies to sensitize IB-R.

## Materials and methods

### Cell lines and patient samples

Human leukemic cell lines MEC-1 (CLL) and ABC-DLBCL (RIVA and TMD8) (obtained from Dr. Neetu Gupta, Cleveland Clinic) were cultured in RPMI-1640 medium supplemented with 10% FBS (Atlanta Biologicals, Lawrenceville, GA), and antibiotic-antimycotic (Gibco, Life Technologies, Gaithersburg, MD). Ibrutinib-R cells (IB-R) were cultured with 5% FBS. Cell lines were routinely screened for *Mycoplasma*, variations in growth rates, changes in morphological characteristics, and their response to stress with Annexin V FITC-PI staining; their passage number did not exceed 20. Ibrutinib was obtained from Santa Cruz (Chicago, IL); Selinexor (KPT-330) and idelalisib (GS-1101) from Selleck Chemicals (Houston, TX).

Peripheral blood samples were obtained from CLL patients after informed consent according to protocols approved by the institutional review board (IRB) according to the Declaration of Helsinki. Briefly, lymphocytes from blood samples were purified by Ficoll-Paque PLUS (Amersham Biosciences, Piscataway, NJ) gradient centrifugation. Primary cells were cultured and RNA was extracted using the TRIZOL reagent (Life Technologies) according to the manufacturer’s instructions.

### Generation of ibrutinib-resistant (IB-R) cell lines

Ibrutinib-resistant MEC-1, RIVA and TMD8 cells were generated by in vitro culture of the parental cell lines for prolonged periods of time with progressively increasing concentrations of ibrutinib. Briefly, cells were intermittently incubated with a low concentration (five-fold lower than IC_50_) of ibrutinib for short intervals over time and allowed to recover after washing off the drug. The ibrutinib concentration and treatment time were gradually increased until cells remained viable after a continuous exposure to the drug that was double the concentration of their IC_50_ value. The IB-R cells were routinely tested for resistance to ibrutinib and cultured without drug for 72 h before they were used in experiments. DNA sequencing was performed by GENEWIZ for ibrutinib-resistant MEC-1 and RIVA cells for BTK C481S, PLCG2 (R665W, S707Y and L845F) mutations. Briefly, end-sequencing was performed utilizing the following primers PLCG2-ex-19-F AAGCCCACTGTGTGATTTCC, PLCG2-ex-19-R TTTTGCTGTGGTGGTTGTTG, PLCG2-ex-20-F TTGAAAATACCCACATCATTGTTT, PLCG2-ex-20-R TATCCCCAGGACCTACAGCA, PLCG2-ex-24-F TCTCTCCCCATGGACGTATC, PLCG2-ex-24-R ACTCACTGACCAGCCTGACC, BTK-ex-15-F ACTCCTAGGTCAGCCCCTTC, BTK-ex-15-R TGGAGCTGAGGCTGGAGATA. After enzymatic purification, sequencing was performed in-house by using BigDye Terminator Cycle Sequencing. Double strand sequencing was provided.

### Cell viability assay

The number of viable cells in culture was determined by 3-(4,5-dimethylthiazol-2-yl)-5-(3-carboxymethoxyphenyl)-2-(4-sulfophenyl)-2H tetrazolium inner salt (MTS) assay (Promega, Madison, WI) and the percentage reduction in metabolic activity was calculated as previously described^8^.

### Apoptosis assay

The percentage of cells undergoing apoptosis was measured by phosphatidlyserine externalization using fluorescein conjugated Annexin V/PI double staining (BD Biosciences, San Jose, CA). The analysis was done on a BD FACS MACSQuant flow cytometer (BD Biosciences), and the raw data was processed using the FlowJo software. The results were normalized to survival of control cells that have been treated with DMSO.

### Immunoblotting

To prepare lysates, cells were collected and washed twice with ice-cold PBS. Cell lysates were prepared in RIPA buffer (20 mM Tris (pH 7.5), 150 mM NaCl, 1 mM EDTA, 1 mM EGTA, 1% Triton X-100, 1% phosphatase inhibitor cocktail (Sigma) and 1 mM PMSF (Sigma) for 30-45 min at 4 °C. Specifically, nuclear/cytoplasmic fractions were prepared using lysis buffer A (20 mM Tris pH 8.0, 10 mM NaCl, 3 mM MgC_l2_, 0.1% NP-40, 10% glycerol, 0.2 mM EDTA with 1% phosphatase inhibitor cocktail and 1 mM PMSF) and lysis buffer C (20 mM Tris pH 8.0, 400 mM NaCl, 0.2 mM EDTA, 20% glycerol, 1 mM DTT with 1% phosphatase inhibitor cocktail and 1 mM PMSF) at 4 °C. The protein concentration in each sample was determined using the Bradford reagent (Bio-Rad, Hercules, CA, USA); 50 µg protein was resolved on 10% SDS-PAGE followed by transferring to nitrocellulose membrane (Millipore, Danvers, MA). The immunoblotting was performed with primary antibodies for BIM (BD Biosciences) Cat No. 559685, PTEN (Cascade Bioscience) Cat No. ABM-2052, cleaved caspase 3 Cat No. #9661 S, FOXO3a (75D8) Cat No. #2497 S, pFOXO3a^Ser253^ Cat No. #9466, AKT Cat No. #9272, pAKT^Ser473^ Cat No. #9271 S (Cell Signaling Technologies, Danvers, MA), GAPDH (G-9) Cat no. sc-365062 (Santa Cruz Biotechnology, Santa Cruz, CA), and β-actin (Sigma) Cat No. A5441. The secondary HRP-conjugated anti-mouse Cat No. 31450 and -rabbit Cat No. 31460 antibodies were purchased from Thermo Fisher Scientific (Pittsburgh, PA). The immunoreactive bands were visualized by chemiluminescence according to the manufacturer’s recommendations (Thermo Fisher, Waltham, MA).

### RNA isolation and real-time quantitative-PCR

RNA was extracted using the TRIZOL reagent (Life Technologies) from parental and IB-R cells after ibrutinib treatment according to the manufacturer’s instructions. Levels of mRNA were analyzed using a quantitative real-time reverse transcriptase PCR (qRT-PCR) kit (Life Technologies), with primers for *pten*, *bim* and *foxo3a*, and normalized for *β-actin*, as described. Primers were for *pten* (forward: 5ʹ-CCAATGTTCAGTGGCGGAACT-3ʹ; reverse: 5ʹ-GAACTTGTCTTCCCGTCGTGTG-3ʹ), *foxo3a* (forward: 5ʹ-CGGACAAACGGCTCACTCT-3ʹ; reverse: 5′-GGACCCGCATGAATCGACTAT-3ʹ), *bim* (forward: 5ʹ-TGGCAAAGCAACCTTCTGATG-3ʹ; reverse: 5ʹ-GCAGGCTGCAATTGTCTACCT-3ʹ) and *β-actin* (forward: 5ʹ-AGAAAATCTGGCACCACACC-3ʹ; reverse: 5ʹ-AGAGGCGTACAG-GGATAGCA-3ʹ) were synthesized by IDT^R^. The band intensities of each band were normalized to the corresponding *β-actin* bands.

### siRNA transfection

FOXO3a, PTEN, BIM and AKT knockdown was achieved using specific siRNA or siControl (Santa Cruz Biotechnology) by the AMAXA Nucleofector Kit V (Lonza, Walkersville, MD) according to the manufacturer’s protocol.

### Chromatin Immunoprecipitation assay (ChIP) assay

ChIP assays were performed using the EZ-ChIP^TM^ Chromatin IP Kit (Millipore) according to the manufacturer’s instructions. Briefly, the proteins were crosslinked to DNA using 1% formaldehyde (Sigma-Aldrich). The crosslinked chromatin was extracted from cells using buffer A and buffer B provided in the ChIP kit and then digested using micrococcal nuclease for 20 min at 37 °C into small fragments. The crosslinked chromatin was aliquoted and immunoprecipitated using 2 μg of Foxo3a antibody and normal rabbit immunoglobulin G antibody overnight at 4 °C with gentle rotation. The complexes were captured by ChIP-Grade Protein G agarose beads for 2 h at 4 °C with gentle rotation. Following thorough washing, bound DNA fragments were eluted and analyzed by subsequent PCR using primers for *pten* and *bim* as follows: *pten* (forward: 5ʹ-GCATTTCCGAATCAGCTCTCT-3ʹ; reverse: 5ʹ-CCAAGTGACTTATCTCTGGTCTGAG-3ʹ) and *bim* (forward: 5ʹ-AGGCAGAACAGGAGGAGA-3ʹ; reverse: 5ʹ-AACCCGTTTGTAAGAGGC-3ʹ).

### Statistical analysis

Each experiment was repeated at least three times. For all the quantitative analyses represented in the graphs, the values are expressed as the mean values ± S.D. The significance of the differences between mean values were assessed using 2–tailed Student’s *t*-test and One-way ANOVA with Bonferroni’s Multiple Comparison Test was performed using GraphPad Prism Version 5.00.

## Supplementary information

Supplementary Table 1

Supplementary Table 2

Supplemental Figure Legends

Supplementary Fig. S1

Supplementary Fig. S2

Supplementary Fig. S3

Supplementary Fig. S4

Supplementary Fig. S5

## References

[1] Furman RR (2014). Ibrutinib resistance in chronic lymphocytic leukemia. N. Engl. J. Med..

[2] Roschewski M, Staudt LM, Wilson WH (2014). Diffuse large B-cell lymphoma-treatment approaches in the molecular era. Nat. Rev. Clin. Oncol..

[3] Cultrera JL, Dalia SM (2012). Diffuse large B-cell lymphoma: current strategies and future directions. Cancer Control.

[4] Anderson MA, Tsui A, Wall M, Huang DC, Roberts AW (2016). Current challenges and novel treatment strategies in double hit lymphomas. Ther. Adv. Hematol..

[5] Wiestner A (2013). Targeting B-Cell receptor signaling for anticancer therapy: the Bruton's tyrosine kinase inhibitor ibrutinib induces impressive responses in B-cell malignancies. J. Clin. Oncol..

[6] Woyach JA (2014). Resistance mechanisms for the Bruton's tyrosine kinase inhibitor ibrutinib. N. Engl. J. Med..

[7] Albitar A (2017). Using high-sensitivity sequencing for the detection of mutations in BTK and PLCgamma2 genes in cellular and cell-free DNA and correlation with progression in patients treated with BTK inhibitors. Oncotarget.

[8] Choudhary GS (2015). MCL-1 and BCL-xL-dependent resistance to the BCL-2 inhibitor ABT-199 can be overcome by preventing PI3K/AKT/mTOR activation in lymphoid malignancies. Cell Death Dis..

[9] Reddy A (2017). Genetic and functional drivers of diffuse large B cell lymphoma. Cell.

[10] Wang J (2017). AKT hyperactivation and the potential of AKT-targeted therapy in diffuse large B-cell lymphoma. Am. J. Pathol..

[11] Ma Y (2015). Evaluation of AKT phosphorylation and PTEN loss and their correlation with the resistance of rituximab in DLBCL. Int. J. Clin. Exp. Pathol..

[12] Alfieri R, Giovannetti E, Bonelli M, Cavazzoni A (2017). New treatment opportunities in phosphatase and tensin homolog (PTEN)-deficient tumors: focus on PTEN/focal adhesion kinase pathway. Front. Oncol..

[13] Wang X (2018). Clinical significance of PTEN deletion, mutation, and loss of PTEN expression in de novo diffuse large B-cell lymphoma. Neoplasia.

[14] Farhan M (2017). FOXO signaling pathways as therapeutic targets in cancer. Int. J. Biol. Sci..

[15] Obrador-Hevia A, Serra-Sitjar M, Rodriguez J, Villalonga P, Fernandez de Mattos S (2012). The tumour suppressor FOXO3 is a key regulator of mantle cell lymphoma proliferation and survival. Br. J. Haematol..

[16] Xie M (2019). Akt2 mediates glucocorticoid resistance in lymphoid malignancies through FoxO3a/Bim axis and serves as a direct target for resistance reversal. Cell Death Dis..

[17] Zhang SQ, Smith SM, Zhang SY, Lynn Wang Y (2015). Mechanisms of ibrutinib resistance in chronic lymphocytic leukaemia and non-Hodgkin lymphoma. Br. J. Haematol..

[18] Mondello P (2018). Panobinostat acts synergistically with ibrutinib in diffuse large B cell lymphoma cells with MyD88 L265P mutations. JCI Insight.

[19] Kadri S (2017). Clonal evolution underlying leukemia progression and Richter transformation in patients with ibrutinib-relapsed CLL. Blood Adv..

[20] Landau DA (2017). The evolutionary landscape of chronic lymphocytic leukemia treated with ibrutinib targeted therapy. Nat. Commun..

[21] Zhang X, Tang N, Hadden TJ, Rishi AK (2011). Akt, FoxO and regulation of apoptosis. Biochim. Biophys. Acta.

[22] Luo H (2013). PTEN-regulated AKT/FoxO3a/Bim signaling contributes to reactive oxygen species-mediated apoptosis in selenite-treated colorectal cancer cells. Cell Death Dis..

[23] Shen WH (2007). Essential role for nuclear PTEN in maintaining chromosomal integrity. Cell.

[24] Zhong Y (2014). Selinexor suppresses downstream effectors of B-cell activation, proliferation and migration in chronic lymphocytic leukemia cells. Leukemia.

[25] Hing ZA (2015). Selinexor is effective in acquired resistance to ibrutinib and synergizes with ibrutinib in chronic lymphocytic leukemia. Blood.

[26] Noorolyai S, Shajari N, Baghbani E, Sadreddini S, Baradaran B (2019). The relation between PI3K/AKT signalling pathway and cancer. Gene.

[27] Zhao X (2017). Unification of de novo and acquired ibrutinib resistance in mantle cell lymphoma. Nat. Commun..

[28] Kornblau SM (2010). Highly phosphorylated FOXO3A is an adverse prognostic factor in acute myeloid leukemia. Clin. Cancer Res..

[29] Santo EE (2013). FOXO3a is a major target of inactivation by PI3K/AKT signaling in aggressive neuroblastoma. Cancer Res..

[30] Habashy HO (2011). FOXO3a nuclear localisation is associated with good prognosis in luminal-like breast cancer. Breast Cancer Res. Treat..

[31] Sharma A (2013). BECN1 and BIM interactions with MCL-1 determine fludarabine resistance in leukemic B cells. Cell Death Dis..

[32] Zhang L (2017). B-cell lymphoma patient-derived xenograft models enable drug discovery and are a platform for personalized therapy. Clin. Cancer Res..

[33] Pfeifer M (2013). PTEN loss defines a PI3K/AKT pathway-dependent germinal center subtype of diffuse large B-cell lymphoma. Proc. Natl Acad. Sci. USA.

[34] Yap TA, Bjerke L, Clarke PA, Workman P (2015). Drugging PI3K in cancer: refining targets and therapeutic strategies. Curr. Opin. Pharmacol..

[35] Bodo J (2013). The phosphatidylinositol 3-kinases (PI3K) inhibitor GS-1101 synergistically potentiates histone deacetylase inhibitor-induced proliferation inhibition and apoptosis through the inactivation of PI3K and extracellular signal-regulated kinase pathways. Br. J. Haematol..

[36] Erdmann T (2017). Sensitivity to PI3K and AKT inhibitors is mediated by divergent molecular mechanisms in subtypes of DLBCL. Blood.

[37] Yang Q, Modi P, Newcomb T, Queva C, Gandhi V (2015). Idelalisib: first-in-class PI3K delta inhibitor for the treatment of chronic lymphocytic leukemia, small lymphocytic leukemia, and follicular lymphoma. Clin. Cancer Res..

[38] Madanat YF, Smith MR, Almasan A, Hill BT (2016). Idelalisib therapy of indolent B-cell malignancies: chronic lymphocytic leukemia and small lymphocytic or follicular lymphomas. Blood Lymphat Cancer.

[39] Visentin A (2019). BCR kinase inhibitors, idelalisib and ibrutinib, are active and effective in Richter syndrome. Br. J. Haematol..

[40] Davids MS (2019). Umbralisib in combination with ibrutinib in patients with relapsed or refractory chronic lymphocytic leukaemia or mantle cell lymphoma: a multicentre phase 1-1b study. Lancet Haematol..

[41] Lapalombella R (2012). Selective inhibitors of nuclear export show that CRM1/XPO1 is a target in chronic lymphocytic leukemia. Blood.

[42] Cerchietti LC (2010). A small-molecule inhibitor of BCL6 kills DLBCL cells in vitro and in vivo. Cancer Cell..

