# Supplementary figures and images for: Resistance to BTK inhibition by ibrutinib can be overcome by preventing FOXO3a nuclear export and PI3K/AKT activation in B-cell lymphoid malignancies
Source: Cell Death Dis. 2019 Dec 4;10(12):924. doi: 10.1038/s41419-019-2158-0 (PMC6892912; doi:10.1038/s41419-019-2158-0)

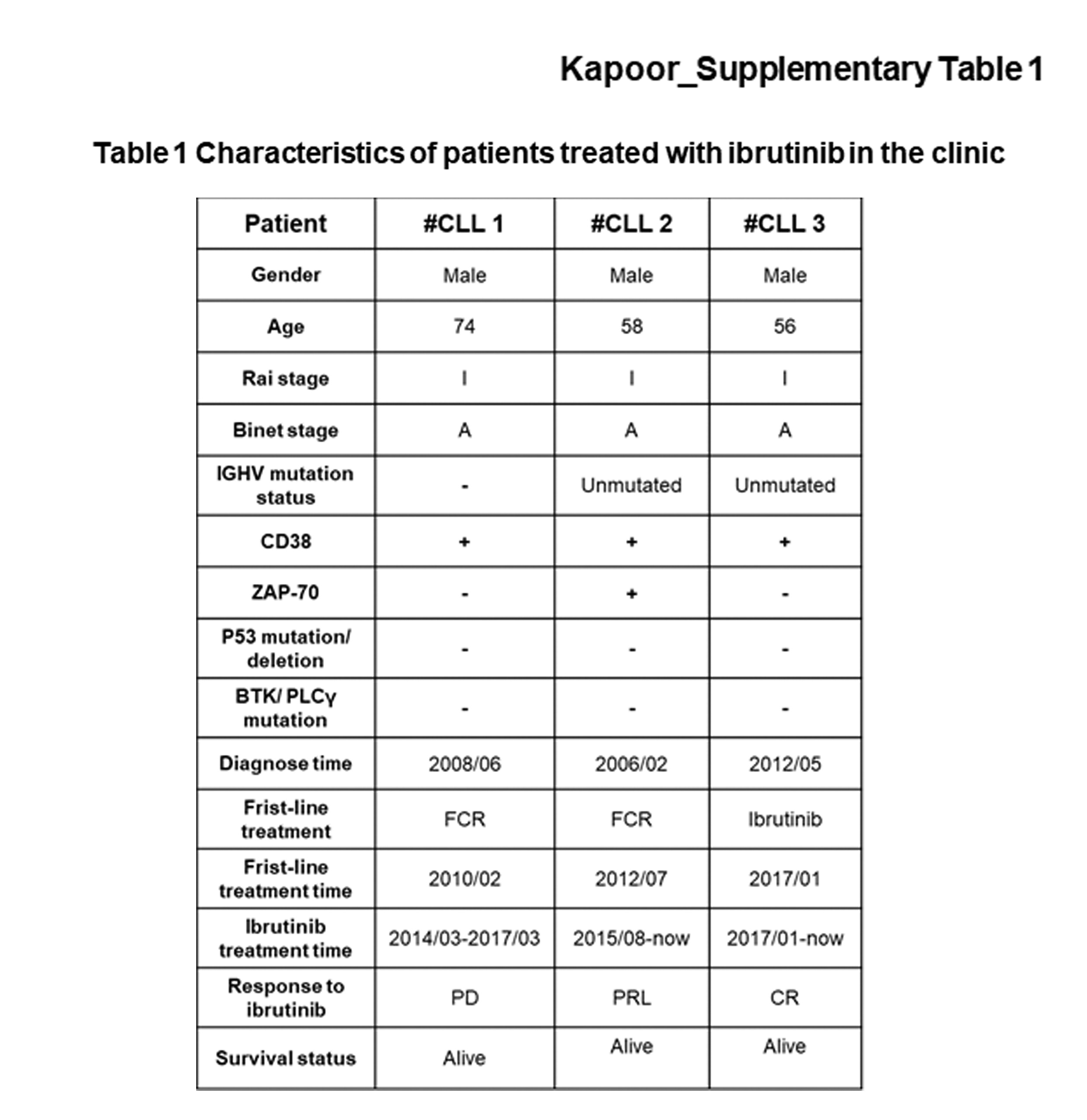

Supplement: Supplementary file 1 — Supplementary Table 1 [file 41419_2019_2158_MOESM1_ESM.tif]

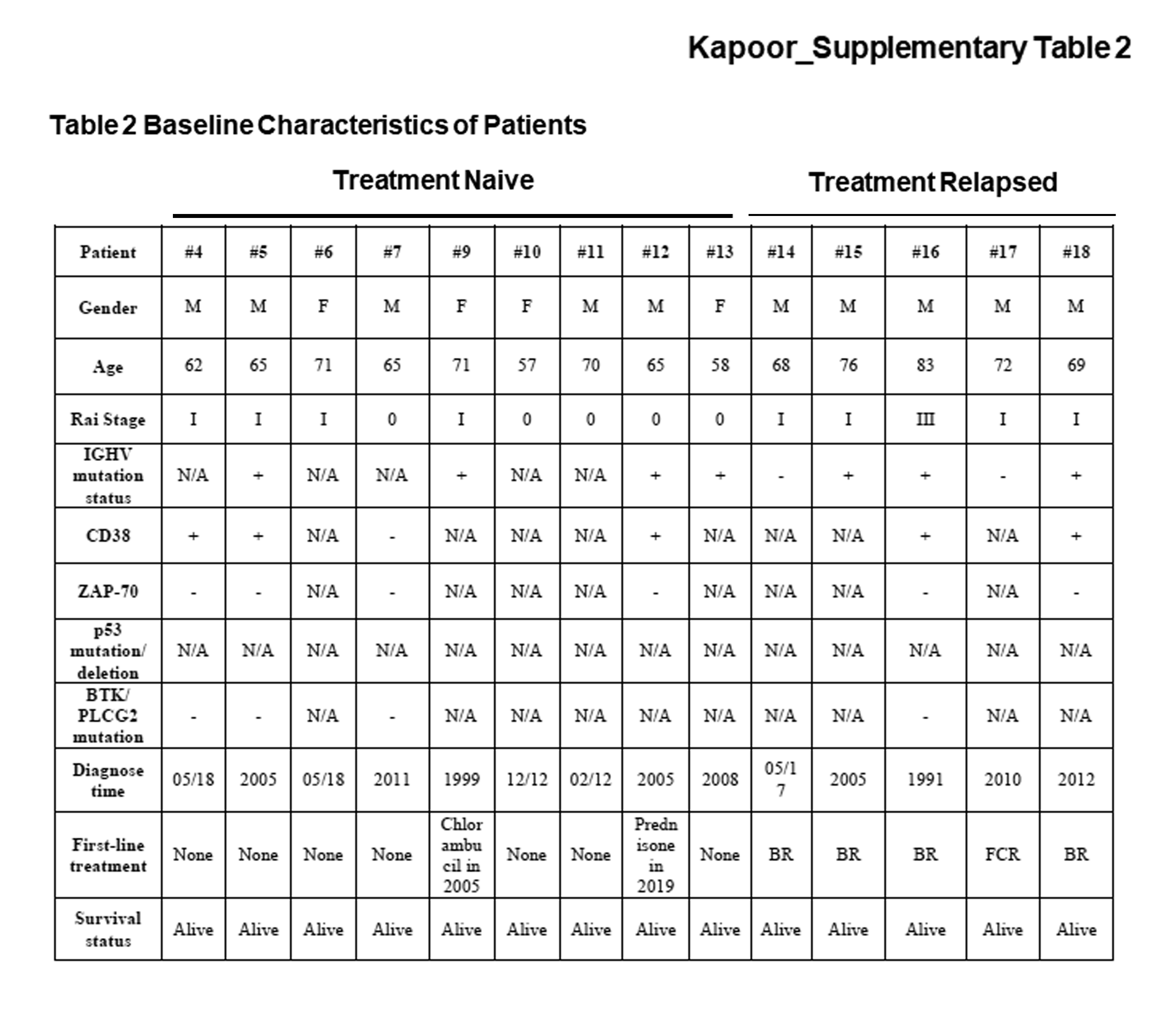

Supplement: Supplementary file 2 — Supplementary Table 2 [file 41419_2019_2158_MOESM2_ESM.tif]

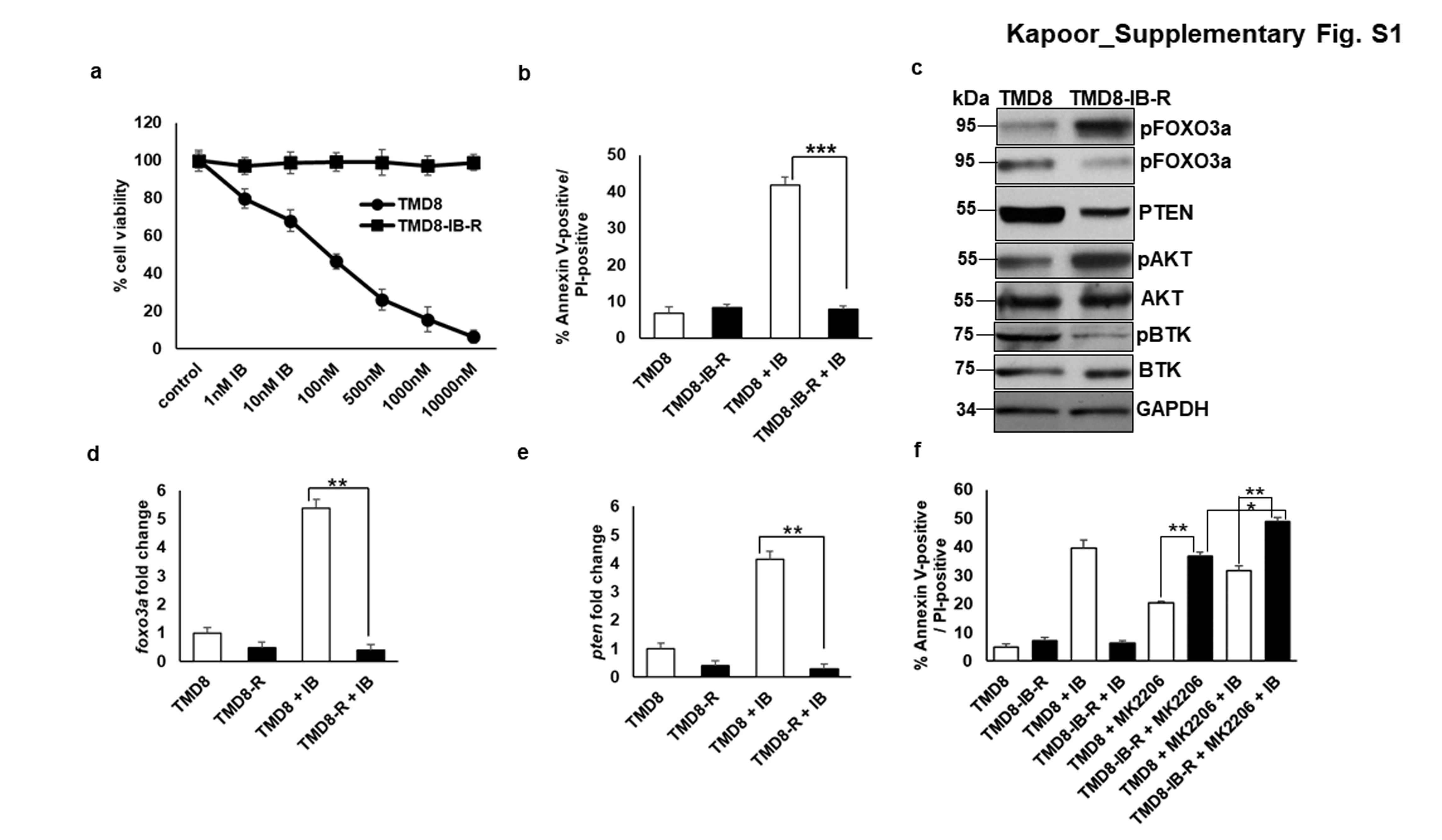

Supplement: Supplementary file 4 — Supplementary Fig. S1 [file 41419_2019_2158_MOESM4_ESM.tif]

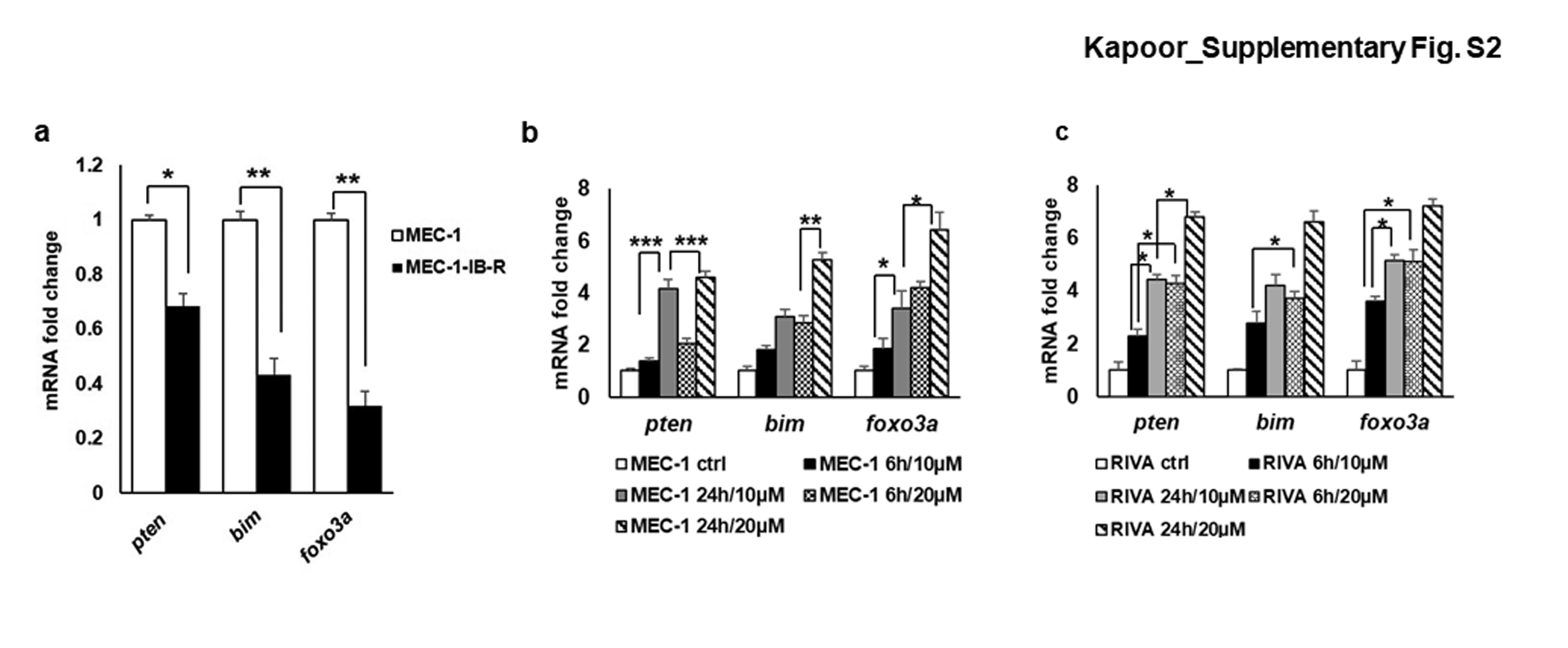

Supplement: Supplementary file 5 — Supplementary Fig. S2 [file 41419_2019_2158_MOESM5_ESM.tif]

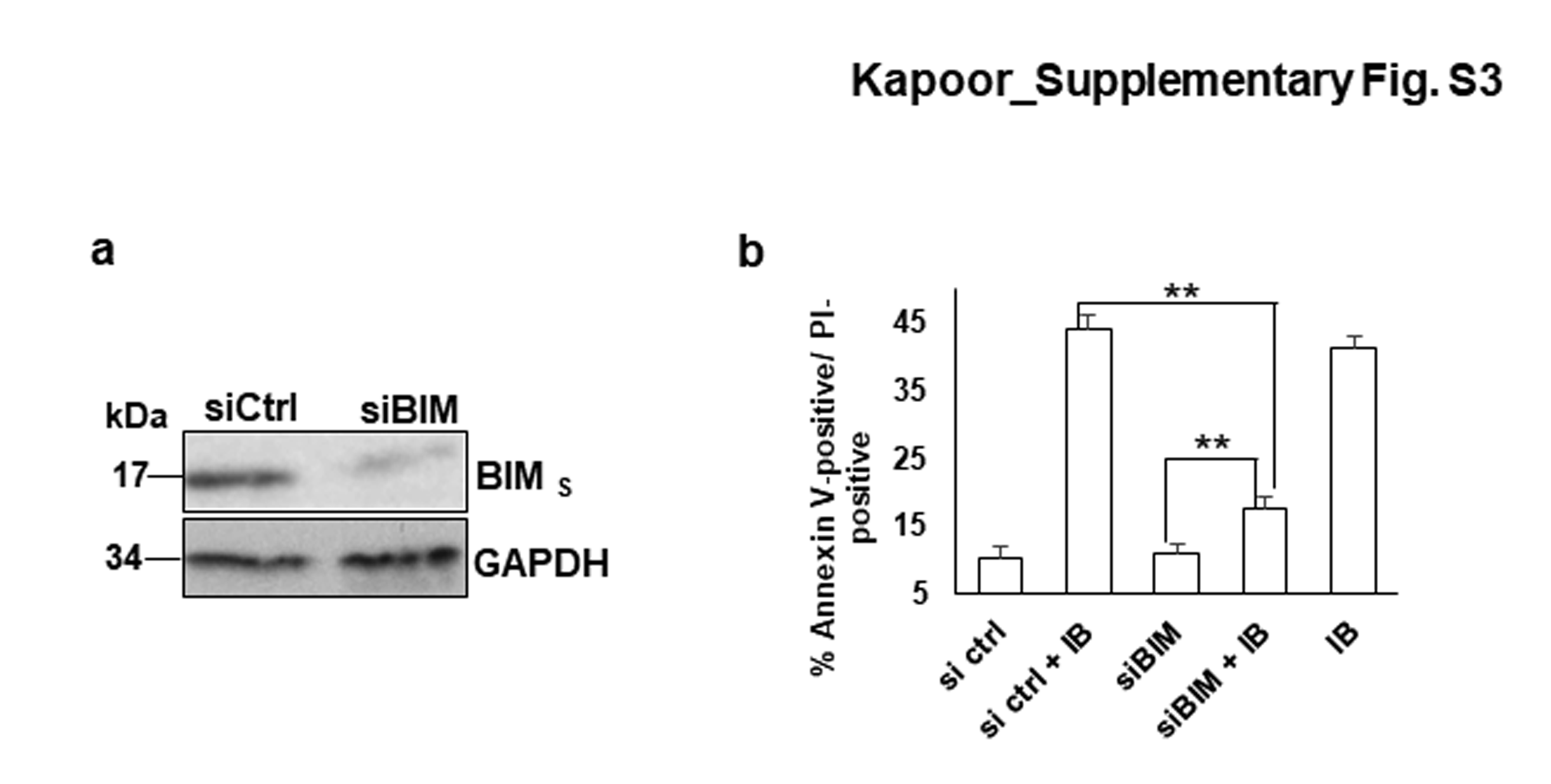

Supplement: Supplementary file 6 — Supplementary Fig. S3 [file 41419_2019_2158_MOESM6_ESM.tif]

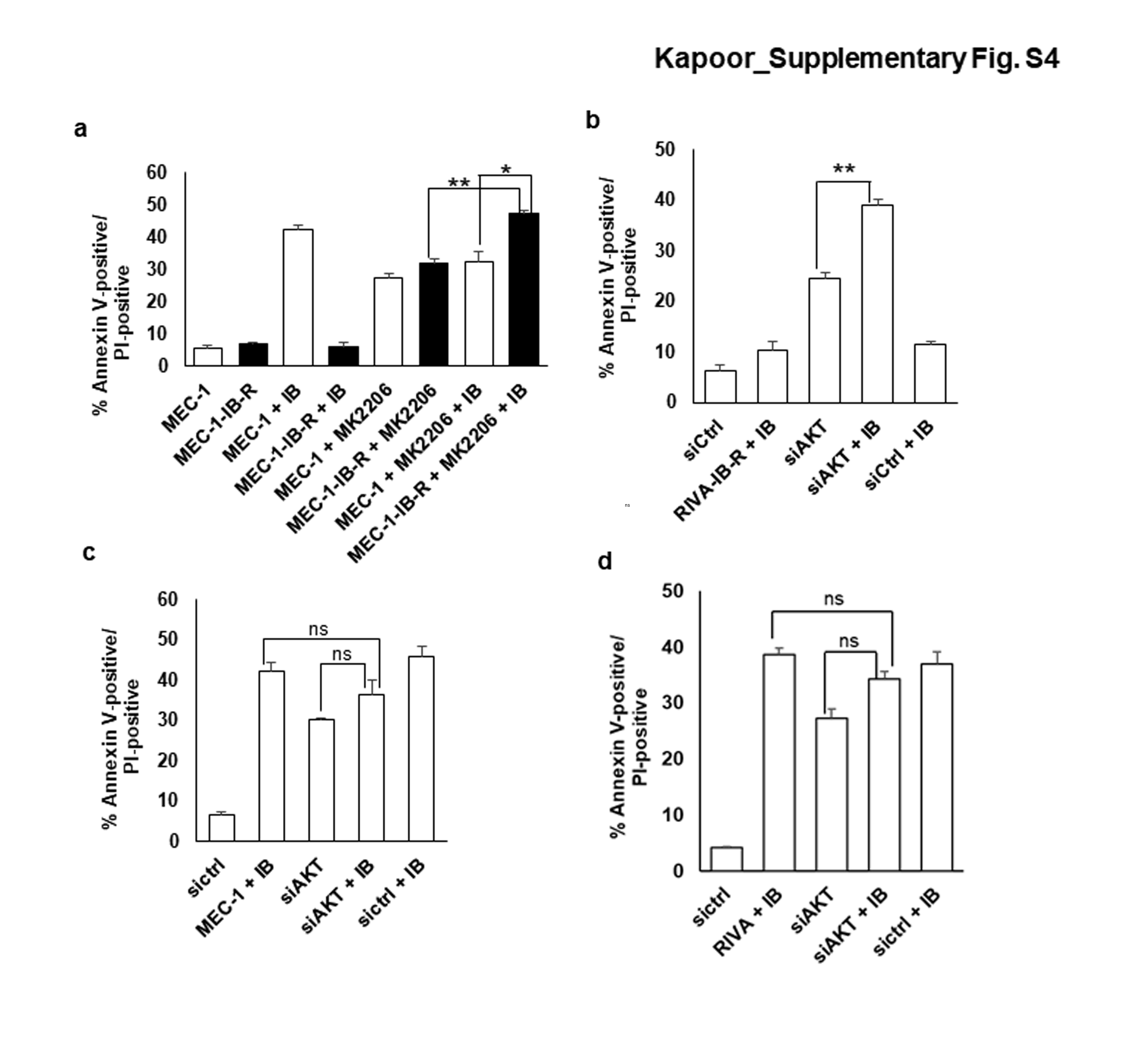

Supplement: Supplementary file 7 — Supplementary Fig. S4 [file 41419_2019_2158_MOESM7_ESM.tif]

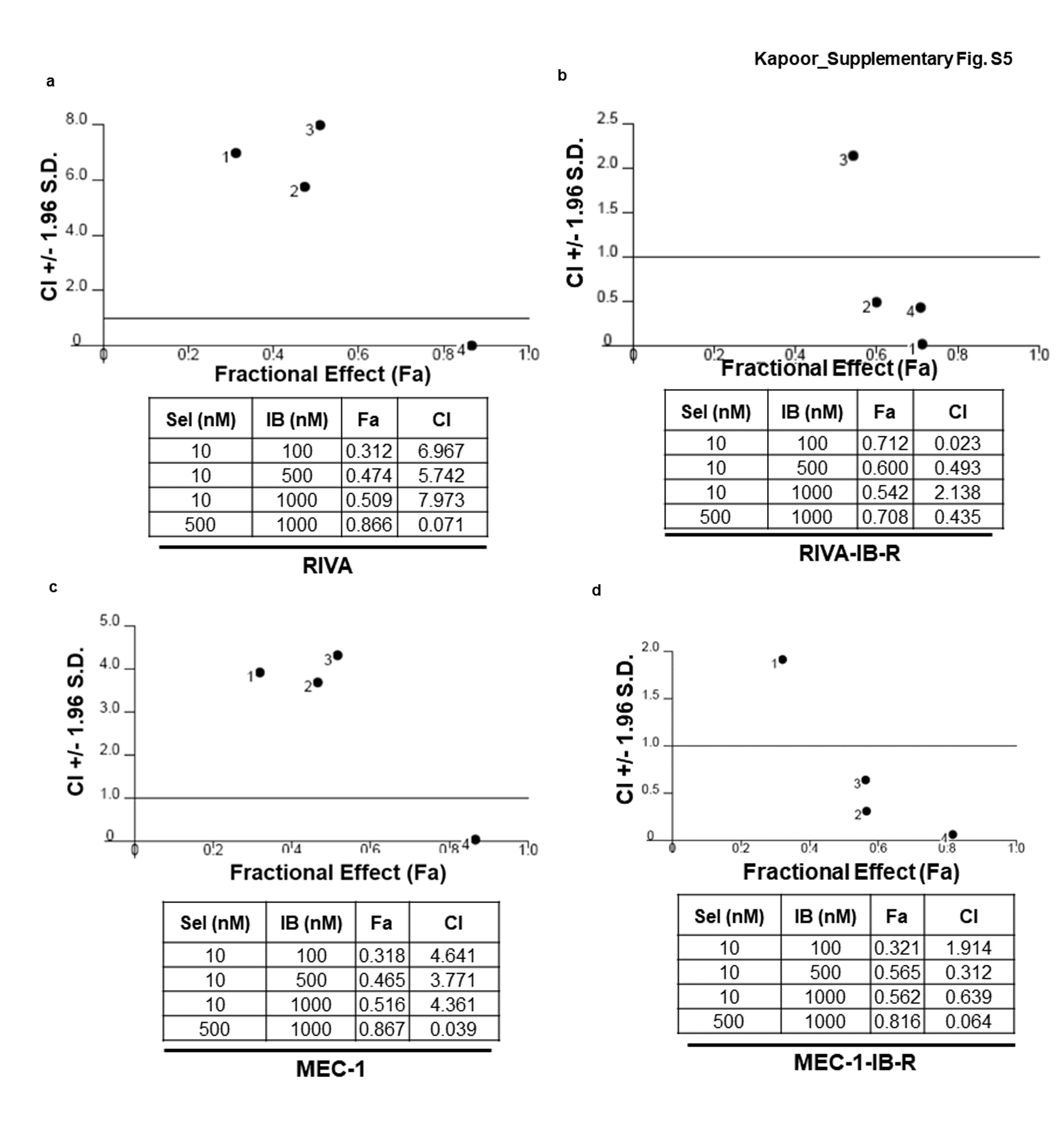

Supplement: Supplementary file 8 — Supplementary Fig. S5 [file 41419_2019_2158_MOESM8_ESM.tif]
