# Supplementary material for: Resistance to BTK inhibition by ibrutinib can be overcome by preventing FOXO3a nuclear export and PI3K/AKT activation in B-cell lymphoid malignancies
Source: Cell Death Dis. 2019 Dec 4;10(12):924. doi: 10.1038/s41419-019-2158-0 (PMC6892912; doi:10.1038/s41419-019-2158-0)
Supplement: Supplementary file 3 — Supplemental Figure Legends [file 41419_2019_2158_MOESM3_ESM.doc]

**Supplemental Figure Legends**

**Supplementary Fig. S1 Acquired resistance to ibrutinib leads to decreased FOXO3a and PTEN levels and activation of AKT in TMD8 cells a** TMD8 cells were treated with the indicated concentrations of ibrutinib for 72 h and cell viability was determined by the MTS assay. Control cells were treated with DMSO. **b** Cell death analysis in parental TMD8 and ibrutinib-resistant derivative (TMD8-1IB-R) in response to 24 h ibrutinib treatment determined by Annexin-V/PI staining. All data are expressed as mean + S.D. of percentage of cell death. Standard deviation (SD) is indicated as error bars (*N=3*). **c** Expression levels of pFOXO3a^Ser253^, FOXO3a, pAKT^Ser473^, AKT and PTEN in whole cell extracts of untreated parental and IB-R TMD8 and TMD8 cell lines. GAPDH was used as a loading control. **d, e** mRNA fold change of *foxo3a* and *pten* in parental *vs.* IB-R TMD8 cells with or without ibrutinib (10 µM). **f** TMD8 and TMD8-IB-R cells were treated with MK2206 (5 µM) and ibrutinib (10 µM) either alone or in combination for 24 h. Cell viability was determined by Annexin V-PI staining. Control cells were treated with DMSO. *(*p<0.05, **p<0.01*). SD is indicated as error bars (*N=3*).

**Supplementary Fig. S2 Ibrutinib treatment regulates transcriptional activation of *pten*, *bim* and *foxo3a* in CLL and ABC-DLBCL cells a** mRNA fold change of *pten*, *bim,* and *foxo3a* in parental *vs.* IB-R MEC-1 cells after culture in the absence of ibrutinib for 72 h. *(*p<0.05, **p<0.01*). Standard deviation (SD) is indicated as error bars (*N=3*). **b, c** *pten*, *bim* and *foxo3a* mRNA fold dose- and time-dependent change was analyzed in parental MEC-1 and RIVA cells after ibrutinib treatment. *(*p<0.05, **p<0.01, ***p<0.001*). SD is indicated as error bars (*N=3*).

**Supplementary Fig. S3 Ibrutinib induces apoptosis in BIM-dependent manner a** RIVA cells were transfected with siBIM and siControl and treated with ibrutinib (10 µM) for 24 h. Expression levels of BIM, was determined by immunoblotting. GAPDH was used as a loading control. **b** Cell viability was determined by Annexin V-PI staining. Control cells were treated with DMSO. *(**p<0.01*). SD is indicated as error bars (*N=3*).

**Supplementary Fig. S4 AKT inhibition increases ibrutinib-induced apoptosis in IB-R cells a** MEC-1 and MEC-1-IB-R cells were treated with MK2206 (5 µM) or ibrutinib (10 µM), either alone or in combination for 24 h. Cell viability was determined by Annexin V-PI staining. Control cells were treated with DMSO. *(*p<0.05, **p<0.01*). SD is indicated as error bars (*N=3*). **b** RIVA-IB-R cells were transfected with siAKT and siControl and treated with ibrutinib (10 µM) for 24 h. Cell viability was determined by Annexin V-PI staining. Control cells were treated with DMSO. *(^ns^p>0.05, **p<0.01*). SD is indicated as error bars (*N=3*). **c, d** MEC-1 and RIVA cells were transfected with siAKT and siControl and treated with ibrutinib (10 µM) for 24 h. Cell viability was determined by Annexin V-PI staining. Control cells were treated with DMSO. *(^ns^p>0.05).* SD is indicated as error bars (*N=3*).

**Supplementary Fig. S5 Selinexor synergizes with ibrutinib in RIVA-IB-R and MEC-1-IB-R cells**

**a, b** Fractional-effect/ combination index curves were generated with the Chou-Talalay method of statistical analysis in RIVA and RIVA-IB-R cells (upper panel). Cells were treated for 48 h with the indicated doses. The summarized combination index values (CI) for apoptosis were generated for RIVA and RIVA-IB-R cells (lower panel). **c, d** Fractional-effect/ combination index curves were generated with the Chou-Talalay method of statistical analysis in MEC-1 and MEC-1-IB-R cells (upper panel). Cells were treated for 48 h with the indicated doses. The summarized combination index values (CI) for apoptosis were generated for MEC-1 and MEC-1-IB-R cells (lower panel).
